# 1-Ethyl-5-iodo­indoline-2,3-dione

**DOI:** 10.1107/S1600536813033539

**Published:** 2013-12-14

**Authors:** Lei Wang, Yu-Xiang Shen, Jian-Tong Dong, Man Zhang, Qi Fang

**Affiliations:** a School of Chemistry and Chemical Engineering, Shandong University, Jinan 250100, People’s Republic of China; b State Key Laboratory of Crystal Materials, Shandong University, Jinan 250100, People’s Republic of China

## Abstract

There are two independent mol­ecules in the asymmetric unit of the title compound, C_10_H_8_INO_2_, which differ in the degree of planarity. The iodo­indoline-2,3-dione skeleton of mol­ecule 1 is essentially planar [mean deviation = 0.003 (2) Å for the nine non-H atoms of the indoline core, with a maximum deviation of 0.033 (1) Å for the I atom]. The I atom and O atom in the 3-position of mol­ecule 2 deviate by 0.195 (1) and 0.120 (2) Å, respectively, from the least-squares plane through the nine non-H atoms of the indoline core. Mol­ecules 1 and 2 are roughly coplanar, the mean planes through their cores making a dihedral angle of 6.84 (1)°. This coplanarity results in a layer-like structure parallel to (6,11,17) in the crystal, the distance between adjacent least-squares planes through the cores of mol­ecules 1 and 2 being 3.37 (1) Å. In such a layer, mol­ecules 1 and 2 are linked by C—H⋯O hydrogen bonds, forming chains along [11-1]. The chains are further coupled to construct a kind of double-chain structure *via* I⋯O inter­actions [3.270 (2) Å].

## Related literature   

For applications of indoline-2,3-dione in drug design, see: Silva *et al.* (2001 ▶). For the synthesis of the title compound, see: Ji *et al.* (2010 ▶). For related structures, see: Garden *et al.* (2006 ▶); Abid *et al.* (2008 ▶); Kurkin *et al.* (2008 ▶).
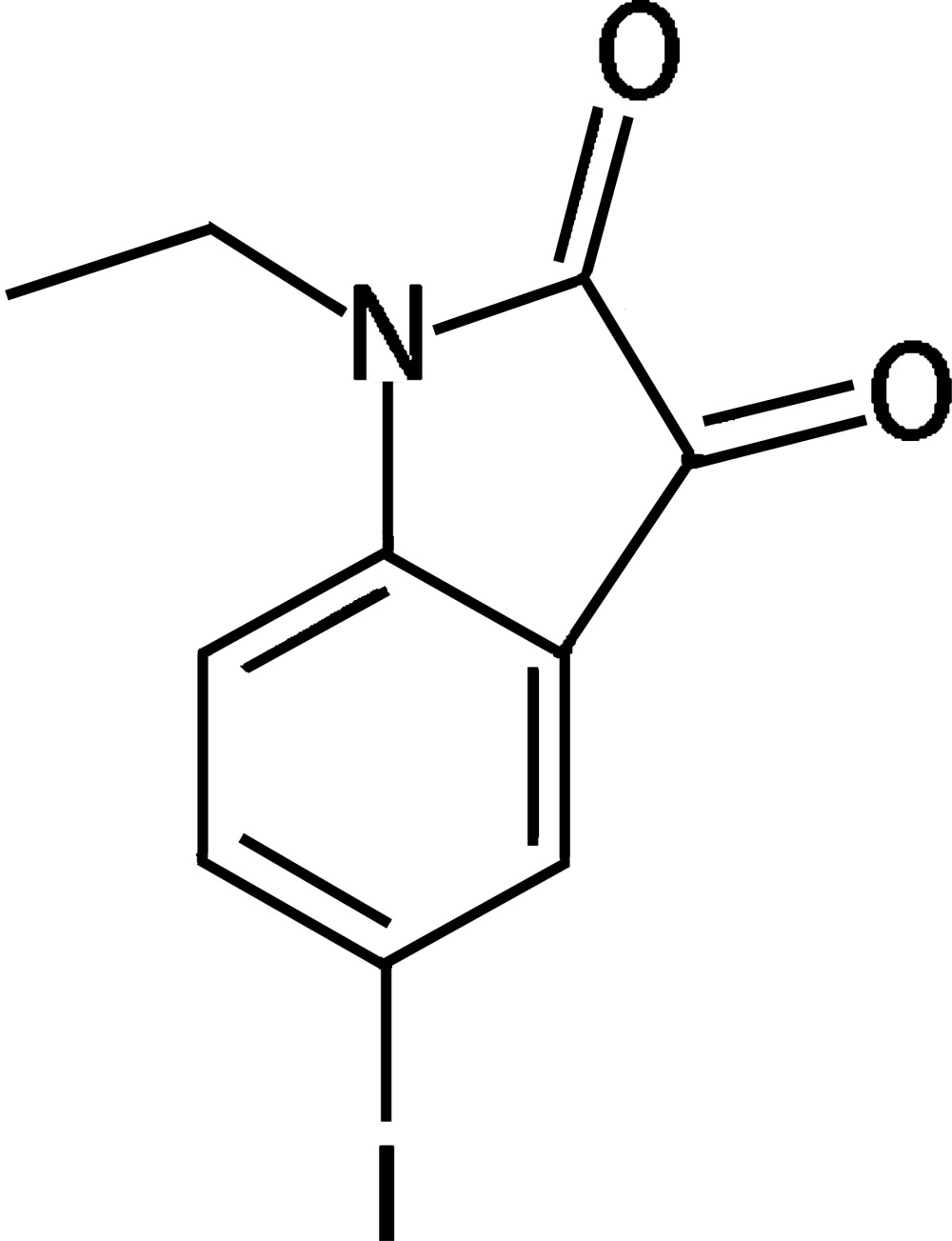


## Experimental   

### 

#### Crystal data  

C_10_H_8_INO_2_
*M* *_r_* = 301.07
Triclinic, 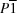

*a* = 9.9658 (2) Å
*b* = 10.1453 (2) Å
*c* = 11.3007 (2) Å
α = 71.188 (1)°
β = 72.599 (1)°
γ = 84.434 (1)°
*V* = 1032.04 (3) Å^3^
*Z* = 4
Mo *K*α radiation
μ = 3.08 mm^−1^
*T* = 295 K
0.27 × 0.21 × 0.10 mm

#### Data collection  

Bruker APEXII CCD diffractometer
Absorption correction: multi-scan (*SADABS*; Bruker, 2005 ▶) *T*_min_ = 0.490, *T*_max_ = 0.746
13515 measured reflections
5091 independent reflections
4358 reflections with *I* > 2σ(*I*)
*R*_int_ = 0.017

#### Refinement  

*R*[*F* ^2^ > 2σ(*F* ^2^)] = 0.023
*wR*(*F* ^2^) = 0.066
*S* = 1.01
5091 reflections
279 parameters
H atoms treated by a mixture of independent and constrained refinement
Δρ_max_ = 0.74 e Å^−3^
Δρ_min_ = −0.44 e Å^−3^

### 

Data collection: *APEX2* (Bruker, 2005 ▶); cell refinement: *SAINT* (Bruker, 2005 ▶); data reduction: *SAINT*; program(s) used to solve structure: *SHELXS97* (Sheldrick, 2008 ▶); program(s) used to refine structure: *SHELXL97* (Sheldrick, 2008 ▶); molecular graphics: *SHELXTL* (Sheldrick, 2008 ▶) and *Mercury* (Macrae *et al.*, 2006 ▶); software used to prepare material for publication: *SHELXL97*.

## Supplementary Material

Crystal structure: contains datablock(s) I, global. DOI: 10.1107/S1600536813033539/vm2202sup1.cif

Structure factors: contains datablock(s) I. DOI: 10.1107/S1600536813033539/vm2202Isup2.hkl

Supporting information file. DOI: 10.1107/S1600536813033539/vm2202Isup3.cml

Additional supporting information:  crystallographic information; 3D view; checkCIF report

## Figures and Tables

**Table 1:** Hydrogen-bond geometry (Å, °)

*D*—H⋯*A*	*D*—H	H⋯*A*	*D*⋯*A*	*D*—H⋯*A*
C29—H29*A*⋯O2^i^	0.97	2.57	3.399 (3)	144
C27—H27⋯O2^i^	0.93 (3)	2.48 (3)	3.407 (3)	174 (3)
C9—H9*A*⋯O4	0.97	2.56	3.366 (3)	140

Symmetry code: (i) 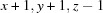.

**Fig. 1.**
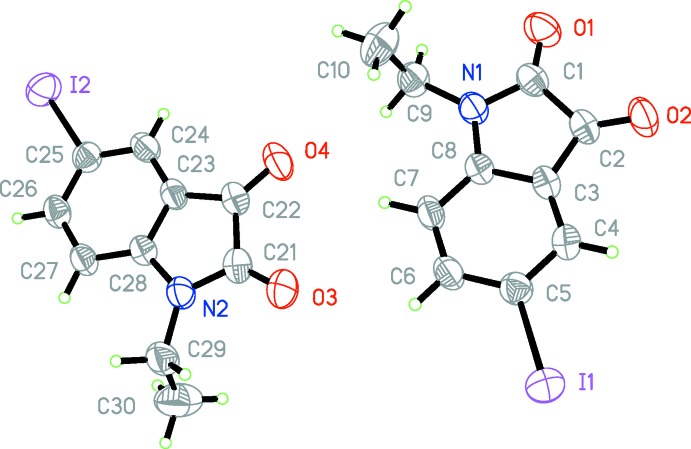
Molecular structure of the two molecules in the asymmetric unit. Displacement ellipsoids are drawn at 50% probability level.

**Fig. 2.**
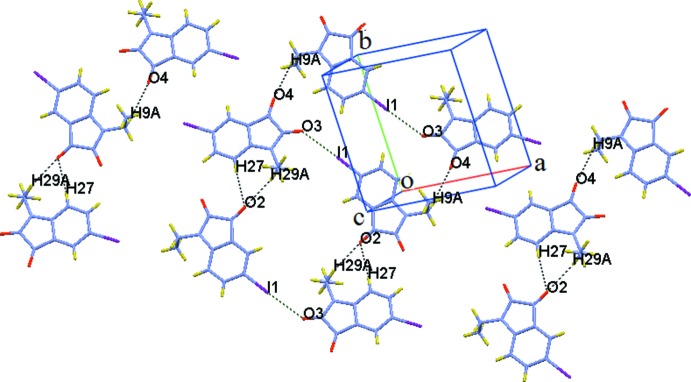
A view of a molecular layer, showing the double-chain structure connected by C—H···O intermolecular hydrogen bonds and I1···O3 intermolecular contacts.

**Fig. 3.**
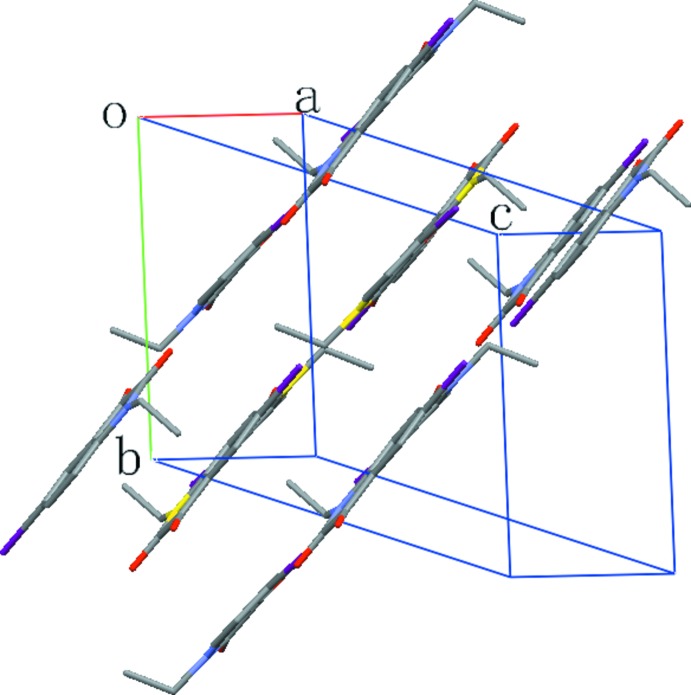
A view of the uniform layered structure, the spacing between neighboring layers is 3.37 (1) Å.

## supplementary crystallographic information

### 1. Comment

To date, isatin and its derivatives have received much attention due to their potential applications in biomedicine and agrochemical industries (Silva *et al.*, 2001). In this paper, we report the synthesis and structure of a new isatin derivative, namely 1-ethyl-5-iodoindoline-2,3-dione.

There are two molecules in the asymmetric unit of the unit cell as depicted in Fig. 1. The two molecules are essentially planar. All non-hydrogen atoms, except the terminal methyl C atom, are in a same plane. The iodoindoline-2,3-dione skeleton of molecule 1 has a perfect planarity (mean deviation is 0.003 (2) Å, maximum deviation is 0.033 (1) Å for I1 for the least-squares plane through the 9 non-hydrogen atoms of the indoline core). In molecule 2, two large deviations exist [0.195 (1) (I2) and 0.120 (2) Å (O3)], and the mean deviation is relatively larger [0.022 (2) Å]. Molecules 1 and 2 are virtually co-planar with a small dihedral angle of 6.84 (1) ° between both best planes. This co-planarity results in a layer-like structure of the crystal (Figs. 2 and 3). Considering this co-planarity, the least-squares plane through the 18 non-hydrogen atoms of the two indoline cores of the two molecules shows a mean deviation of 0.064 (3) Å. The distance between two such adjacent best planes or layers is 3.37 (1) Å.

Several intermolecular interactions can be found in a layer. The C—H···O hydrogen bonds (Table 1) help to build one-dimensional chains and the I1···O3 [-*x*, 1 - *y*, 1 - *z*] [3.270 (2) Å] short contact helps to construct a kind of double-chain structure.

### 2. Experimental

We synthesized the title compound by the similar method reported by Ji *et al.* (2010). KI (1.27 g), hexadecyl trimethyl ammonium bromide (0.410 g) and 5-iodoisatin (3.75 g) were dissolved in 20 ml DMSO, and the mixture was stirred in N_2_ atmosphere while 2.7 ml iodoethane was quickly added *via* a syringe. Then 5 ml aqueous KOH solution (17.8 mol/*L*) was dropwise added into the brown solution at 30 °C and the colour rapidly became black. After another 3 ml iodoethane was added, the heating temperature was raised to 45 °C. The mixture was stirred for 5 h, then 1.6 ml iodoethane was added. The reaction continued for 1 h at 45°C. The mixture was then poured into water, and extracted with dichloromethane. The organic phase was seperated and dried over anhydrous Na_2_SO_4_. The solvent was evaporated under reduced pressure, the crude product was purified by column chromatography [V (dichloromethane)/ V (petroleum ether) = 1:1] obtaining 47.4% yield. Crystals suitable for X-ray diffraction were obtained by slow evaporation of a dichloromethane solution of the compound.

### 3. Refinement

H atoms bound to aromatic C atoms were located in difference maps and freely refined leading to C—H distances from 0.88 (3) to 0.99 (3) Å. Other H atoms were placed at calculated positions and treated by the riding model with C—H distances = 0.97 (methylene C) or 0.96 Å (methyl C) and *U*_iso_(H) = 1.2 *U*_eq_ (methylene C) or 1.5 *U*_eq_ (methyl C).

### Crystal data

**Table:**

C_10_H_8_INO_2_	*Z* = 4
*M**_r_* = 301.07	*F*(000) = 576
Triclinic, *P*1	*D*_x_ = 1.938 Mg m^−^^3^
Hall symbol: -P 1	Mo *K*α radiation, λ = 0.71073 Å
*a* = 9.9658 (2) Å	Cell parameters from 7943 reflections
*b* = 10.1453 (2) Å	θ = 2.4–28.7°
*c* = 11.3007 (2) Å	µ = 3.08 mm^−^^1^
α = 71.188 (1)°	*T* = 295 K
β = 72.599 (1)°	Plank, orange
γ = 84.434 (1)°	0.27 × 0.21 × 0.10 mm
*V* = 1032.04 (3) Å^3^	

### Data collection

**Table:**

Bruker APEXII CCD diffractometer	5091 independent reflections
Radiation source: fine-focus sealed tube	4358 reflections with *I* > 2σ(*I*)
Graphite monochromator	*R*_int_ = 0.017
Detector resolution: 8.3 pixels mm^-1^	θ_max_ = 28.3°, θ_min_ = 2.0°
φ and ω scans	*h* = −13→13
Absorption correction: multi-scan (*SADABS*; Bruker, 2005)	*k* = −13→13
*T*_min_ = 0.490, *T*_max_ = 0.746	*l* = −15→13
13515 measured reflections	

### Refinement

**Table:**

Refinement on *F*^2^	Primary atom site location: structure-invariant direct methods
Least-squares matrix: full	Secondary atom site location: difference Fourier map
*R*[*F*^2^ > 2σ(*F*^2^)] = 0.023	Hydrogen site location: mixed
*wR*(*F*^2^) = 0.066	H atoms treated by a mixture of independent and constrained refinement
*S* = 1.01	*w* = 1/[σ^2^(*F*_o_^2^) + (0.0323*P*)^2^ + 0.529*P*] where *P* = (*F*_o_^2^ + 2*F*_c_^2^)/3
5091 reflections	(Δ/σ)_max_ = 0.001
279 parameters	Δρ_max_ = 0.74 e Å^−^^3^
0 restraints	Δρ_min_ = −0.44 e Å^−^^3^

### Special details

**Table:**

Experimental. Scan width 0.5° ω and φ, Crystal to detector distance 5.964 cm, exposure time 20 s, 19 h for data collection
Geometry. All e.s.d.'s (except the e.s.d. in the dihedral angle between two l.s. planes) are estimated using the full covariance matrix. The cell e.s.d.'s are taken into account individually in the estimation of e.s.d.'s in distances, angles and torsion angles; correlations between e.s.d.'s in cell parameters are only used when they are defined by crystal symmetry. An approximate (isotropic) treatment of cell e.s.d.'s is used for estimating e.s.d.'s involving l.s. planes.
Refinement. Refinement of *F*^2^ against ALL reflections. The weighted *R*-factor *wR* and goodness of fit *S* are based on *F*^2^, conventional *R*-factors *R* are based on *F*, with *F* set to zero for negative *F*^2^. The threshold expression of *F*^2^ > σ(*F*^2^) is used only for calculating *R*-factors(gt) *etc*. and is not relevant to the choice of reflections for refinement. *R*-factors based on *F*^2^ are statistically about twice as large as those based on *F*, and *R*- factors based on ALL data will be even larger.

### Fractional atomic coordinates and isotropic or equivalent isotropic displacement parameters (Å^2^)

**Table:**

	*x*	*y*	*z*	*U*_iso_*/*U*_eq_	
I1	−0.188160 (18)	0.385210 (17)	0.659889 (17)	0.05692 (6)	
I2	1.215992 (18)	0.17083 (2)	0.320573 (19)	0.06423 (7)	
O1	0.1685 (2)	−0.30982 (18)	0.98576 (18)	0.0606 (5)	
O2	−0.12166 (19)	−0.20187 (18)	1.00153 (18)	0.0587 (4)	
O4	0.5791 (2)	0.1463 (2)	0.5519 (2)	0.0810 (7)	
O3	0.41994 (19)	0.3947 (2)	0.4463 (2)	0.0673 (5)	
N1	0.22397 (19)	−0.09288 (18)	0.83363 (18)	0.0448 (4)	
C29	0.6039 (3)	0.5967 (3)	0.2206 (3)	0.0574 (6)	
H29A	0.6799	0.6622	0.1950	0.069*	
H29B	0.5200	0.6337	0.2693	0.069*	
C5	−0.0444 (2)	0.2289 (2)	0.7122 (2)	0.0451 (5)	
C6	0.0995 (3)	0.2509 (2)	0.6564 (2)	0.0492 (5)	
C7	0.1973 (2)	0.1495 (2)	0.6900 (2)	0.0476 (5)	
C8	0.1470 (2)	0.0250 (2)	0.7828 (2)	0.0408 (4)	
C9	0.3765 (2)	−0.1004 (3)	0.8019 (3)	0.0521 (5)	
H9A	0.4165	−0.0557	0.7092	0.063*	
H9B	0.4054	−0.1973	0.8216	0.063*	
C10	0.4329 (3)	−0.0314 (4)	0.8766 (3)	0.0731 (8)	
H10A	0.3986	0.0625	0.8625	0.110*	
H10B	0.5338	−0.0306	0.8470	0.110*	
H10C	0.4023	−0.0820	0.9679	0.110*	
C1	0.1354 (3)	−0.1967 (2)	0.9239 (2)	0.0453 (5)	
C2	−0.0166 (2)	−0.1390 (2)	0.9316 (2)	0.0444 (5)	
C3	0.0021 (2)	0.0023 (2)	0.8392 (2)	0.0409 (4)	
C4	−0.0948 (2)	0.1026 (2)	0.8047 (2)	0.0441 (5)	
C25	1.0250 (2)	0.2682 (2)	0.3052 (2)	0.0456 (5)	
C24	0.9007 (3)	0.2058 (2)	0.3914 (2)	0.0492 (5)	
C23	0.7765 (2)	0.2774 (2)	0.3829 (2)	0.0439 (5)	
C28	0.7764 (2)	0.4083 (2)	0.2908 (2)	0.0404 (4)	
N2	0.63970 (19)	0.4636 (2)	0.30430 (19)	0.0467 (4)	
C30	0.5792 (4)	0.5831 (4)	0.1017 (3)	0.0877 (11)	
H30A	0.6629	0.5489	0.0519	0.132*	
H30B	0.5553	0.6724	0.0498	0.132*	
H30C	0.5034	0.5191	0.1265	0.132*	
C22	0.6307 (3)	0.2473 (3)	0.4615 (2)	0.0536 (6)	
C21	0.5455 (2)	0.3764 (3)	0.4059 (2)	0.0514 (5)	
C27	0.9001 (2)	0.4696 (2)	0.2032 (2)	0.0461 (5)	
C26	1.0245 (2)	0.3975 (2)	0.2122 (2)	0.0468 (5)	
H4	−0.189 (3)	0.089 (3)	0.843 (2)	0.046 (6)*	
H6	0.132 (3)	0.339 (3)	0.596 (3)	0.055 (7)*	
H7	0.299 (3)	0.166 (3)	0.650 (3)	0.050 (7)*	
H26	1.110 (3)	0.439 (3)	0.149 (3)	0.055 (7)*	
H27	0.897 (3)	0.557 (3)	0.142 (3)	0.052 (7)*	
H24	0.903 (3)	0.124 (3)	0.449 (3)	0.055 (7)*	

### Atomic displacement parameters (Å^2^)

**Table:**

	*U*^11^	*U*^22^	*U*^33^	*U*^12^	*U*^13^	*U*^23^
I1	0.05293 (10)	0.05269 (10)	0.06295 (11)	0.00741 (7)	−0.02024 (8)	−0.01363 (8)
I2	0.04713 (10)	0.07268 (13)	0.07074 (12)	0.01030 (8)	−0.01917 (8)	−0.02004 (9)
O1	0.0694 (12)	0.0412 (9)	0.0596 (10)	0.0013 (8)	−0.0147 (9)	−0.0037 (8)
O2	0.0541 (10)	0.0492 (9)	0.0564 (10)	−0.0162 (8)	−0.0007 (8)	−0.0034 (8)
O4	0.0520 (11)	0.0725 (13)	0.0778 (14)	−0.0168 (10)	−0.0029 (10)	0.0214 (11)
O3	0.0382 (9)	0.0733 (13)	0.0721 (12)	−0.0045 (8)	−0.0035 (8)	−0.0080 (10)
N1	0.0424 (10)	0.0391 (9)	0.0449 (10)	−0.0014 (7)	−0.0058 (8)	−0.0079 (8)
C29	0.0451 (13)	0.0429 (12)	0.0678 (16)	0.0045 (10)	−0.0062 (11)	−0.0056 (11)
C5	0.0458 (12)	0.0415 (11)	0.0449 (11)	0.0021 (9)	−0.0113 (9)	−0.0113 (9)
C6	0.0475 (12)	0.0407 (11)	0.0457 (12)	−0.0062 (9)	−0.0040 (9)	−0.0016 (9)
C7	0.0402 (11)	0.0448 (11)	0.0442 (11)	−0.0079 (9)	−0.0003 (9)	−0.0040 (9)
C8	0.0409 (10)	0.0389 (10)	0.0373 (10)	−0.0038 (8)	−0.0053 (8)	−0.0093 (8)
C9	0.0407 (11)	0.0510 (12)	0.0557 (13)	0.0068 (10)	−0.0043 (10)	−0.0152 (11)
C10	0.0457 (14)	0.097 (2)	0.086 (2)	0.0070 (14)	−0.0178 (14)	−0.0434 (18)
C1	0.0527 (13)	0.0380 (10)	0.0417 (11)	−0.0038 (9)	−0.0080 (9)	−0.0115 (9)
C2	0.0485 (12)	0.0391 (10)	0.0400 (10)	−0.0090 (9)	−0.0041 (9)	−0.0097 (9)
C3	0.0411 (11)	0.0373 (10)	0.0394 (10)	−0.0070 (8)	−0.0045 (8)	−0.0098 (8)
C4	0.0380 (11)	0.0454 (11)	0.0442 (11)	−0.0059 (9)	−0.0036 (9)	−0.0136 (9)
C25	0.0407 (11)	0.0471 (11)	0.0472 (11)	0.0010 (9)	−0.0094 (9)	−0.0151 (9)
C24	0.0499 (13)	0.0404 (11)	0.0476 (12)	−0.0034 (9)	−0.0104 (10)	−0.0027 (10)
C23	0.0414 (11)	0.0421 (11)	0.0403 (10)	−0.0076 (8)	−0.0052 (8)	−0.0058 (9)
C28	0.0401 (11)	0.0375 (10)	0.0388 (10)	−0.0039 (8)	−0.0059 (8)	−0.0094 (8)
N2	0.0378 (9)	0.0440 (9)	0.0481 (10)	−0.0031 (7)	−0.0045 (8)	−0.0067 (8)
C30	0.087 (2)	0.101 (3)	0.0606 (18)	0.025 (2)	−0.0243 (16)	−0.0097 (18)
C22	0.0448 (12)	0.0535 (13)	0.0496 (13)	−0.0112 (10)	−0.0068 (10)	−0.0017 (10)
C21	0.0404 (12)	0.0558 (13)	0.0505 (12)	−0.0079 (10)	−0.0050 (9)	−0.0115 (11)
C27	0.0435 (12)	0.0392 (11)	0.0445 (11)	−0.0066 (9)	−0.0032 (9)	−0.0047 (9)
C26	0.0394 (11)	0.0457 (11)	0.0464 (11)	−0.0057 (9)	−0.0011 (9)	−0.0108 (9)

### Geometric parameters (Å, º)

**Table:**

I1—C5	2.092 (2)	C10—H10A	0.9600
I2—C25	2.086 (2)	C10—H10B	0.9600
O1—C1	1.209 (3)	C10—H10C	0.9600
O2—C2	1.198 (3)	C1—C2	1.558 (3)
O4—C22	1.213 (3)	C2—C3	1.467 (3)
O3—C21	1.215 (3)	C3—C4	1.378 (3)
N1—C1	1.371 (3)	C4—H4	0.91 (3)
N1—C8	1.411 (3)	C25—C24	1.384 (3)
N1—C9	1.454 (3)	C25—C26	1.392 (3)
C29—N2	1.459 (3)	C24—C23	1.386 (3)
C29—C30	1.486 (5)	C24—H24	0.88 (3)
C29—H29A	0.9700	C23—C28	1.399 (3)
C29—H29B	0.9700	C23—C22	1.460 (3)
C5—C4	1.391 (3)	C28—C27	1.381 (3)
C5—C6	1.391 (3)	C28—N2	1.406 (3)
C6—C7	1.388 (3)	N2—C21	1.358 (3)
C6—H6	0.95 (3)	C30—H30A	0.9600
C7—C8	1.378 (3)	C30—H30B	0.9600
C7—H7	0.99 (3)	C30—H30C	0.9600
C8—C3	1.401 (3)	C22—C21	1.551 (4)
C9—C10	1.503 (4)	C27—C26	1.391 (3)
C9—H9A	0.9700	C27—H27	0.93 (3)
C9—H9B	0.9700	C26—H26	0.96 (3)
			
C1—N1—C8	110.78 (18)	C4—C3—C8	121.64 (19)
C1—N1—C9	124.24 (19)	C4—C3—C2	131.1 (2)
C8—N1—C9	124.86 (18)	C8—C3—C2	107.30 (19)
N2—C29—C30	112.1 (2)	C3—C4—C5	117.8 (2)
N2—C29—H29A	109.2	C3—C4—H4	121.8 (16)
C30—C29—H29A	109.2	C5—C4—H4	120.4 (16)
N2—C29—H29B	109.2	C24—C25—C26	121.0 (2)
C30—C29—H29B	109.2	C24—C25—I2	119.38 (17)
H29A—C29—H29B	107.9	C26—C25—I2	119.59 (17)
C4—C5—C6	120.3 (2)	C25—C24—C23	117.7 (2)
C4—C5—I1	118.94 (17)	C25—C24—H24	119.6 (18)
C6—C5—I1	120.75 (16)	C23—C24—H24	122.7 (18)
C7—C6—C5	122.0 (2)	C24—C23—C28	121.3 (2)
C7—C6—H6	118.8 (17)	C24—C23—C22	132.1 (2)
C5—C6—H6	119.2 (17)	C28—C23—C22	106.5 (2)
C8—C7—C6	117.6 (2)	C27—C28—C23	121.1 (2)
C8—C7—H7	121.0 (16)	C27—C28—N2	127.6 (2)
C6—C7—H7	121.5 (16)	C23—C28—N2	111.23 (18)
C7—C8—C3	120.7 (2)	C21—N2—C28	110.83 (18)
C7—C8—N1	128.4 (2)	C21—N2—C29	124.6 (2)
C3—C8—N1	110.93 (18)	C28—N2—C29	124.59 (18)
N1—C9—C10	112.0 (2)	C29—C30—H30A	109.5
N1—C9—H9A	109.2	C29—C30—H30B	109.5
C10—C9—H9A	109.2	H30A—C30—H30B	109.5
N1—C9—H9B	109.2	C29—C30—H30C	109.5
C10—C9—H9B	109.2	H30A—C30—H30C	109.5
H9A—C9—H9B	107.9	H30B—C30—H30C	109.5
C9—C10—H10A	109.5	O4—C22—C23	130.6 (3)
C9—C10—H10B	109.5	O4—C22—C21	124.0 (2)
H10A—C10—H10B	109.5	C23—C22—C21	105.38 (19)
C9—C10—H10C	109.5	O3—C21—N2	127.6 (2)
H10A—C10—H10C	109.5	O3—C21—C22	126.4 (2)
H10B—C10—H10C	109.5	N2—C21—C22	105.98 (19)
O1—C1—N1	126.9 (2)	C28—C27—C26	117.4 (2)
O1—C1—C2	127.0 (2)	C28—C27—H27	119.1 (17)
N1—C1—C2	106.10 (18)	C26—C27—H27	123.5 (17)
O2—C2—C3	130.5 (2)	C27—C26—C25	121.5 (2)
O2—C2—C1	124.6 (2)	C27—C26—H26	116.9 (17)
C3—C2—C1	104.89 (18)	C25—C26—H26	121.6 (17)
			
C4—C5—C6—C7	−0.1 (4)	C26—C25—C24—C23	−0.8 (4)
I1—C5—C6—C7	179.48 (19)	I2—C25—C24—C23	176.44 (18)
C5—C6—C7—C8	−0.7 (4)	C25—C24—C23—C28	−0.2 (4)
C6—C7—C8—C3	0.7 (4)	C25—C24—C23—C22	−175.8 (3)
C6—C7—C8—N1	−179.6 (2)	C24—C23—C28—C27	1.4 (4)
C1—N1—C8—C7	−179.6 (2)	C22—C23—C28—C27	177.9 (2)
C9—N1—C8—C7	4.4 (4)	C24—C23—C28—N2	−177.2 (2)
C1—N1—C8—C3	0.1 (3)	C22—C23—C28—N2	−0.6 (3)
C9—N1—C8—C3	−175.9 (2)	C27—C28—N2—C21	−176.6 (2)
C1—N1—C9—C10	−95.9 (3)	C23—C28—N2—C21	1.9 (3)
C8—N1—C9—C10	79.5 (3)	C27—C28—N2—C29	3.4 (4)
C8—N1—C1—O1	−179.3 (2)	C23—C28—N2—C29	−178.2 (2)
C9—N1—C1—O1	−3.2 (4)	C30—C29—N2—C21	−93.7 (3)
C8—N1—C1—C2	0.2 (2)	C30—C29—N2—C28	86.3 (3)
C9—N1—C1—C2	176.3 (2)	C24—C23—C22—O4	−4.6 (5)
O1—C1—C2—O2	−0.2 (4)	C28—C23—C22—O4	179.4 (3)
N1—C1—C2—O2	−179.7 (2)	C24—C23—C22—C21	175.4 (3)
O1—C1—C2—C3	179.0 (2)	C28—C23—C22—C21	−0.7 (3)
N1—C1—C2—C3	−0.5 (2)	C28—N2—C21—O3	177.6 (3)
C7—C8—C3—C4	−0.1 (3)	C29—N2—C21—O3	−2.3 (4)
N1—C8—C3—C4	−179.8 (2)	C28—N2—C21—C22	−2.2 (3)
C7—C8—C3—C2	179.3 (2)	C29—N2—C21—C22	177.9 (2)
N1—C8—C3—C2	−0.4 (2)	O4—C22—C21—O3	1.9 (5)
O2—C2—C3—C4	−0.9 (4)	C23—C22—C21—O3	−178.0 (3)
C1—C2—C3—C4	179.9 (2)	O4—C22—C21—N2	−178.3 (3)
O2—C2—C3—C8	179.7 (3)	C23—C22—C21—N2	1.7 (3)
C1—C2—C3—C8	0.5 (2)	C23—C28—C27—C26	−1.4 (3)
C8—C3—C4—C5	−0.6 (3)	N2—C28—C27—C26	176.9 (2)
C2—C3—C4—C5	−179.9 (2)	C28—C27—C26—C25	0.4 (4)
C6—C5—C4—C3	0.7 (3)	C24—C25—C26—C27	0.8 (4)
I1—C5—C4—C3	−178.87 (16)	I2—C25—C26—C27	−176.51 (19)

### Hydrogen-bond geometry (Å, º)

**Table:**

*D*—H···*A*	*D*—H	H···*A*	*D*···*A*	*D*—H···*A*
C29—H29*A*···O2^i^	0.97	2.57	3.399 (3)	144
C27—H27···O2^i^	0.93 (3)	2.48 (3)	3.407 (3)	174 (3)
C9—H9*A*···O4	0.97	2.56	3.366 (3)	140

Symmetry code: (i) *x*+1, *y*+1, *z*−1.
